# Pyruvate Kinase M2 Plays a Dual Role on Regulation of the EGF/EGFR Signaling via E-Cadherin-Dependent Manner in Gastric Cancer Cells

**DOI:** 10.1371/journal.pone.0067542

**Published:** 2013-06-28

**Authors:** Le-Yi Wang, Yun-Peng Liu, Li-Gang Chen, Yan-Ling Chen, Li Tan, Jing-Jing Liu, Amarsanaa Jazag, Jian-Lin Ren, Bayasi Guleng

**Affiliations:** 1 Department of Gastroenterology, Zhongshan Hospital affiliated to Xiamen University, Xiamen, Fujian Province, China; 2 Medical College of Xiamen University, Xiamen, Fujian Province, China; 3 National Institute of Medical Research, 3rd General Hospital, Ulaanbaatar, Mongolia; Sapporo Medical University, Japan

## Abstract

**Background and Aims:**

EGFR activation and PKM2 expression are instrumental in tumorigenesis. EGFR activation regulates PKM2 functions in a subcellular compartment-dependent manner and promotes gene transcription and tumor growth. In addition, PKM2 is upregulated in EGFR-induced pathways in glioma malignancies. However, we found that PKM2 could also regulate the activity of the EGF/EGFR signaling pathway in gastric cancer cells. We aimed to define the biological mechanisms for PKM2 in regulating the cell motility and invasion.

**Methods:**

We employed stable transfection with short hairpin RNA to stably silence the expression of PKM2 in the BGC823, SGC7901 and AGS gastric cancer cell lines. The effects of PKM2 in vitro were determined by assessing cell migration and invasion. Immunohistochemical analysis was used to explore the relationship among PKM2 and other proteins.

**Results:**

Our results indicate that the knockdown of PKM2 decreased the activity of E-cadherin and enhanced the EGF/EGFR signaling pathway in the gastric cell lines BGC823 and SGC7901 that were positive for E-cadherin expression. However, in the undifferentiated gastric carcinoma cell line AGS, which lacks E-cadherin expression, PKM2 promoted cell migration and invasion. Immunohistochemical analyses showed that the levels of E-cadherin expression, ERK1/2 phosphorylation, and cytoplasmic PKM2 expression were correlated with each other.

**Conclusion:**

PKM2 may play different roles in differently differentiated gastric cancer cell types, and this finding would be consistent with the previous clinical research. The results of our study reveal an important link between PKM2 and E-cadherin during EGFR-stimulated gastric cancer cell motility and invasion.

## Introduction

Pyruvate kinase (PK) mediates the final rate-limiting step of glycolysis by catalyzing the dephosphorylation of phosphoenolpyruvate (PEP) to pyruvate to yield one molecule of ATP. Mammalian cells have four pyruvate kinase isoenzymes (M1, M2, L, and R), which are selectively expressed in different types of cells and tissues [1]. In mammals, the M1 isoform (PKM1) is expressed in most adult tissues. The M2 isoform (PKM2), an alternatively spliced variant of M1, is expressed during embryonic development [2]. Studies have found that cancer cells exclusively express PKM2 [3], [4]. PKM2 has been shown to be essential for aerobic glycolysis in tumors (Warburg effect). Over the years, significant advancements have been made in understanding the function and regulation of PKM2 as a pyruvate kinase and protein kinase in cancer cells [5]. A recent study confirmed that the PKM2 induced by epidermal growth factor (EGF) translocates into the nucleus of glioblastoma cells, interacts with β-catenin and leads to cyclinD1 expression, which promotes cell proliferation and tumorigenesis [6]. These findings reveal a novel role for PKM2 as a transcriptional coactivator. However, there are some controversies regarding the specificity and potential of PKM2 as an anti-cancer target in cancer therapy. A recent finding revealed that PKM2 expression is strongly correlated with gastric cancer differentiation. Differentiated types of cancers express more PKM2 protein than do the undifferentiated ones. PKM2 was an adverse prognostic factor in signet ring cell gastric cancer [7]. The biological role of PKM2 in different differentiation phases and in the development of gastric cancer needs to be further elucidated.

Previous studies regarding PKM2 have focused on tumor metabolism and tumor growth. There have been only a few reports on tumor metastasis. E-Cadherin plays a critical role in maintaining epithelial integrity, and the loss of E-cadherin affects the adhesive repertoire of a cell [8]. Previous studies [9] in vitro have shown that the loss of E-cadherin in human carcinoma cell lines is associated with poor differentiation and a fibroblastoid morphology. The EGF-dependent activation of the EGFR has been reported to be inhibited in an E-cadherin adhesion-dependent manner, which inhibits the ligand-dependent activation of diverse receptor tyrosine kinases [10]. Our research demonstrated that the knockdown of PKM2 decreased the activity of E-cadherin and enhanced the EGF/EGFR signaling pathway in the cell lines BGC823 and SGC7901 that were positive for E-cadherin expression. However, in the undifferentiated gastric carcinoma cell line AGS, which lacks E-cadherin expression, PKM2 promoted cell migration and invasion. The aim of this study was to elucidate the function and mechanism of PKM2 with regard to cell motility in differently differentiated cell lines.

## Materials and Methods

### Cell Culture, Conditions and Transfection

The human gastric cancer cell lines BGC823 (poorly differentiated, according to the provider) and SGC7901 (moderately differentiated) were cultured in RPMI 1640 medium (HyClone, Logan, UT, USA). The AGS cell line (undifferentiated) was cultured in F12K medium. All cells were cultured in medium containing 10% fetal bovine serum (FBS) (Gibco, Detroit, MI, USA) and 100 IU/mL penicillin-streptomycin at 37°C in a 5% CO_2_ humidified atmosphere. The human gastric cancer cell line AGS was ordered from the American Type Culture Collection (ATCC, USA), and the human gastric cancer cell lines SGC7901 and BGC823 were obtained from China Centre for Type Culture Collection (Shanghai, China). SGC7901, BGC823 and AGS cells were transfected with the siPKM2 (pcPUR+U6-siPKM2) or the PU6 (pcPUR+U6-siRenilla) plasmid using FuGENE HD (Roche, Indianapolis, IN). Puromycin (0.1 µg/ml) was used to screen for stably transfected clones. The expression of the PKM2 protein was examined with Western blot analysis using an antibody against PKM2 to validate the ability of the constructs to inhibit the target gene expression; these experiments were repeated three times. Cell cultures were made quiescent by growing them to confluence, and the medium was replaced with fresh medium containing 0.5% serum for 1 day. EGF with 100 ng/ml final concentration was used for cell stimulation. EGF was obtained from Cell Signaling Technology.

### Stable Knockdown of PKM2 and Over-expression of PKM2

A plasmid containing an RNA interference sequence that targeted the PKM2 gene in BGC823, SGC7901 and AGS cells was constructed. The sense oligo for the siPKM2 sequence was 5′-CACCGCGGCAAGATTTATGTGGAACGTGTGCTGTCCGTTCCACGTAGATCTTGCTGCTTTTT-3′, and the antisense oligo was 5′-GCATAAAAAGCAGCAAGATCTACGTGGAACGGACAGCACACGTTCCACATAAATCTTGCCGC-3′. The BGC-823, SGC-7901 and AGS cells were transfected with pcPUR+U6-siPKM2 or pcPUR+U6-siRenilla (control) and selected as puromycin-resistant clones. Western blot analysis was performed to confirm the PKM2 suppression. A plasmid containing PKM2 cDNA sequence was obtained from Invitrogen. The BGC-823, SGC-7901 and AGS cells were transfected with pcDNA6.0-PKM2 or pcDNA6.0-mock (control) and selected as blasticidin-resistant clones. Western blot analysis was performed to confirm the PKM2 expression.

### Protein Extraction and Western Blot Analysis

Cells were re-suspended in lysis buffer containing a protease inhibitor cocktail, and the extracted proteins were separated using 8-10% SDS–PAGE gels. β-Tubulin was used as a loading control. Antibodies against E-cadherin and p-E-cadherin were obtained from Epitomics. The phospho-EGFR (Tyr1068), phospho-PLCγ1 (Tyr783), phospho-AKT (Ser473), phospho-Gab1 (Tyr627), phospho-c-cbl (Tyr700), and phospho-ERK1/2 (Thr202/Tyr204) antibodies were obtained from Cell Signaling Technology.

### RNA Extraction, Reverse Transcription and Real-time PCR

Total RNA was extracted using the TRIzol reagent (Invitrogen, CA, USA). The samples were then treated with DNase for 15 min at room temperature, and the RNA was further purified using an RNA cleanup kit (Qiagen, CA, USA). The reverse transcription (RT) reaction for the first-strand cDNA synthesis was performed using reverse transcriptase (Bio-Rad) with 2 µg of total RNA. Quantitative RT-PCR analysis was performed with the ABI 7500 (Applied Biosystems), and the gene expression levels for each individual sample were normalized to GAPDH. The mean relative gene expression was determined and differences were calculated using the 2^-ΔΔCt^ method of agarose gel electrophoresis. The RT-PCR primer sequences were as follows: sense 5′- TGTGGGAATCCGACGAATG-3′ and antisense 5′- GTCATATGGTGGAGCTGTGGG-3′ for N-Cadherin; sense 5′- CGGGAATGCAGTTGAGGATC-3′ and antisense 5′- AGGATGGTGTAAGCGATGGC-3′ for E-Cadherin; sense 5′- TGTATGGGGAACTGCTGACA-3′ and antisense 5′- GCGTTCATCCTCATCGAAGT-3′ for MMP7; sense 5′- CGACAGTCAGCCGCATCTT-3′ and antisense 5′-CCCCATGGTGTCTGAGCG-3′ for GAPDH.

### Cell Proliferation Assay

Cell proliferation was measured using the Cell Counting Kit-8 (Dojindo, Kamimashiki-gun, Kumamoto, Japan) according to the manufacturer’s instructions. Briefly, the cells were seeded in four 96-well plates at a density of 2×10^3^ cells/well. One plate was taken out at the same time every day after the cells had adhered to the wells. The absorbance was measured with a microplate reader at a wavelength of 450 nm. All experiments were performed in triplicate.

### Transwell Invasion and Wound Healing Assays

The transwell invasion assays were performed with 8.0-µm-pore inserts in a 24-well transwell plate. The basement membrane was hydrated with 500 µL of serum-free RPMI 1640 or F12K medium 30 min before use. For the invasion assay, the gastric cancer cell lines were added to the upper chamber of a transwell with 0.5 mg/mL collagen type l (BD Bioscience, San Jose, CA)-coated filters. RPMI 1640 or F12K medium with 10% fetal bovine serum and 1% of each antibiotic was added to the lower chamber. The BGC and 7901 cells were incubated for 36 hours. The AGS cells were incubated for 24 hours. The invading cells were quantified after Gentian violet staining. Each experiment was performed in triplicate, and the data were expressed as mean values. The wound-healing assay was performed in 6-well plates. Tumor cells in medium containing 10% FBS were seeded into 6-well plates (Corning, CA). Cell cultures were made quiescent by growing them to confluence, and the medium was replaced with fresh medium containing 0.5% serum for 1 day. Wounds in the monolayer were made with sterile pipette tips. Then EGF with 100 ng/ml final concentration was used for cell stimulation. The cells were then washed with PBS and refreshed with medium containing 10% FBS. Photographs were taken at 0 and 24 h. All experiments were performed in triplicate.

### Ethics Statement

This study was approved by the Ethics Committee (No: 20081012) at the Zhongshan Hospital affiliated with Xiamen University, Xiamen, Fujian Province, China, and we obtained written consent statements from all participants involved in this study.

### Preparation of tissue Samples

Tumor tissue specimens were collected from 15 different gastric cancer patients who underwent curative resection at the Zhongshan Hospital, Xiamen University, Xiamen, China. An independent series of corresponding adjacent noncancerous tissues from 15 of the same patients was collected at the same time. All tissue specimens were collected and divided into two parts immediately after tumor resection. One part was collected into liquid nitrogen and stored at -80°C until RNA and protein extraction. The other part was fixed in 4% formaldehyde and embedded in paraffin for histological analysis (Hematoxylin eosin and IHC staining). All specimens were confirmed by pathological diagnosis (the histopathological diagnosis was based on WHO criteria). All experimental cases were grouped accord-ing to the International Union Against Cancer Tumor-Node-Metastasis (TNM) staging system (revised in 2002).

### Immunohistochemistry

Four-micron-thick paraffin sections were either stained with hematoxylin and eosin (H&E) or analyzed for PKM2, p-ERK1/2 and E-cadherin expression by immunohistochemistry. Immunohistochemistry was performed according to the procedures that were recommended by the manufacturer. The reactions were visualized using diaminobenzidine as a chromogenic substrate. The sections were counterstained using hematoxylin and then cleared and mounted. The mean density (IOD/area) was detected in different positive areas of the 15 human gastric cancer specimens using Image-pro Plus software.

### Statistical Analyses

Statistical analyses were performed using SPSS v13.0 (SPSS, Inc.) software. The Independent-Samples T Test and correlation analysis were used to compare the data. All values are expressed as the means ± SD. The differences were considered statistically significant at *P*<0.05.

## Results

### Depletion of PKM2 Promoted Cell Migration and Invasion in BGC823 and SGC7901 Cells with EGF Stimulation

**Figure 1:**
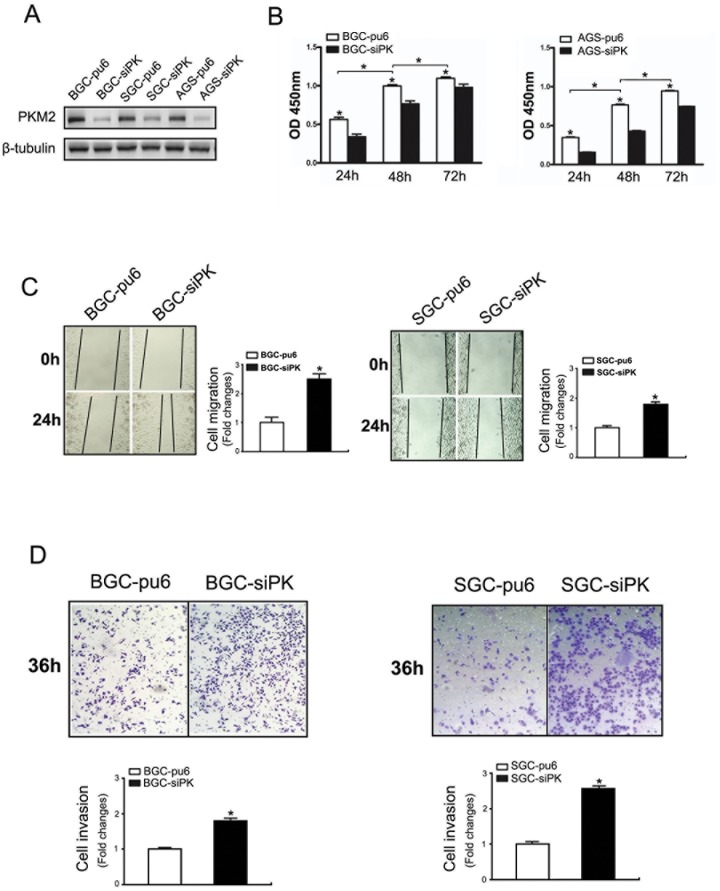
Knockdown of PKM2 promoted the migration and invasion of BGC823 and SGC7901 cells. (A) BGC823, SGC7901 and AGS cells were stably transfected with shRNA directed against PKM2. The specific knockdown of PKM2 was monitored by immunoblot (bottom). Cells stably transfected with pcPUR+U6-siPKM2 are referred to as siPK, and those transfected with pcPUR+U6-siRenilla are referred to as pu6. (B) The proliferation of the stably transfected cells. The cell number was determined with the CCK-8 assay, and the relative number of cells is shown. (C, D) A cross-shaped wound was created in the monolayer, and the BGC823 and SGC7901 stably transfected cells were cultured for an additional 24 hours with EGF (100 ng/ml). Representative images of the wounded region are shown. The results of the migration assay are also shown as graphs (*p<0.05). (E, F) The invasion potential of the BGC823 and SGC7901 stable cells was assessed using the BD transwell chamber assay with 100 ng/ml EGF in the lower chamber for 36 hours. The cells that migrated to the lower side of the filter were stained, photographed, and counted. The data are expressed as the mean ± SD from three independent experiments (*p<0.05).

The expression of the PKM2 protein in the gastric cancer cell lines BGC823, SGC7901 and AGS was evaluated using Western blot analysis. These cell lines showed a high level of PKM2 expression. Then, stable gastric cancer cell lines with an altered PKM2 expression were established using RNA interference (PKM2 knockdown) in the BGC823, SGC7901 and AGS cells. As shown in **Fig. 1A**, the BGC823, SGC7901 and AGS cell lines with PKM2 knockdown were established. We observed that the proliferation was decreased in the BGC823 and AGS cells after PKM2 was depleted (**Fig. 1B**). These results are in agreement with previous studies.

To examine the effect of PKM2 on cell migration and invasion, we employed well-established wound-healing and transwell assays to characterize the cell motility response in the BGC823 and SGC7901 cells. A confluent layer of cells was first incubated overnight in medium, a scratch wound was introduced, medium containing the most suitable dose of EGF (100 ng/ml) was added to stimulate migration, and the percentage of wound sealing was observed after 24 h (Figure S1). The invading cells were quantified 36 h after EGF (100 ng/ml) was added to the lower chamber. The results indicate that the treatment of BGC823-sipk and SGC7901-sipk cells with EGF following a scratch wound and in the transwell significantly increased the rate of wound healing (**Fig. 1 C,D**) and invasion (**Fig. 1 E,F**) compared with that of the control cells.

### Depletion of PKM2 Decreased E-cadherin Expression and Enhanced the Activities of the EGF/EGFR Downstream Signaling Pathways PLC-γ1 and ERK1/2

**Figure 2:**
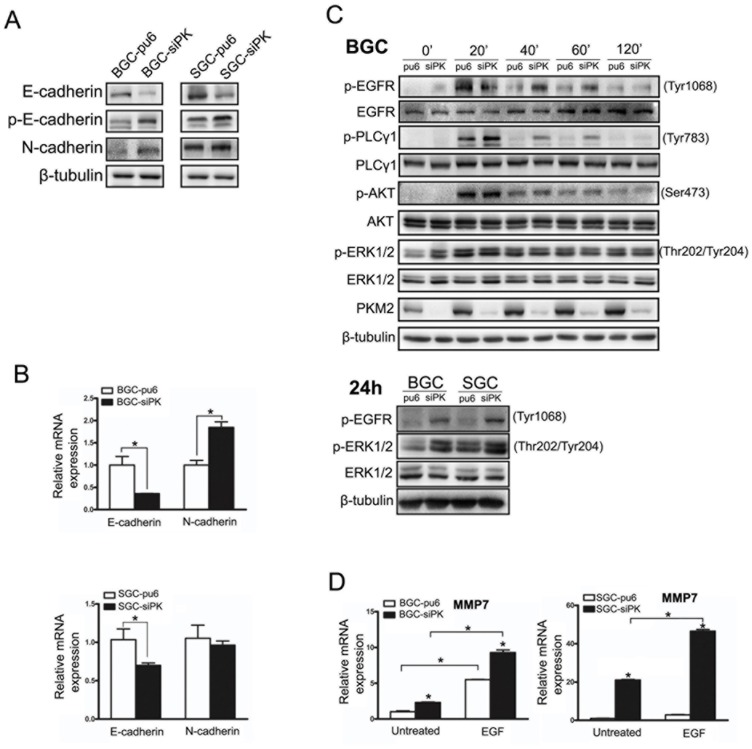
Depletion of PKM2 decreased the expression of E-cadherin and enhanced the activities of the EGF/EGFR downstream signaling pathways. (A) E-cadherin, phospho-E-cadherin and N-cadherin expression levels were analyzed by immunoblot analysis in BGC823 and SGC7901 stable cells. (B) E-cadherin and N-cadherin expression levels were analyzed by quantitative real-time PCR in BGC823 and SGC7901 stable cells. (C) BGC823 and SGC7901 stable cells were exposed to EGF (100 ng/ml) for different times. The Western blots of cell lysates are shown. The phospho-EGFR (Tyr1068), phospho-PLCγ1 (Tyr783), phospho-AKT (ser473), and phospho-ERK1/2 (Thr202/Tyr204) protein levels are shown as indicated. (D) MMP7 expression levels were analyzed by quantitative real-time PCR in BGC823 and SGC7901 stable cells. Error bars represent the mean ± SD of triplicate experiments (*p<0.05).

To investigate the mechanism of changes in cell migration and invasion after the knockdown of PKM2, we analyzed the expression of members of the Ca^2+^-dependent cell adhesion molecules family and metalloproteinases. We found that E-cadherin protein expression levels were decreased in BGC823 and SGC7901 cells when PKM2 expression was reduced (**Fig. 2A**).

The level of E-cadherin mRNA and the phosphorylation of E-cadherin were determined in BGC823 and SGC7901 cells with PKM2 depletion to assess whether the observed difference in E-cadherin expression occurred pre- or post-translationally. We observed the down-regulation of E-cadherin mRNA and increased phosphorylation, which induces the endocytosis of E-cadherin, in PKM2-depleted cells (Fig. 2A, B). We also found that the expression level of the N-cadherin protein was increased in the BGC823 and SGC7901 cell lines when PKM2 was depleted (**Fig. 2A**).

Cell migration and invasion are largely regulated by EGFR activity. To analyze whether the EGFR is involved in the migration and invasion of BGC823 and SGC7901 cells, these cells were treated with EGF, which binds to the EGFR and activates the downstream signaling pathways. The EGF treatment resulted in the phosphorylation of the EGFR and the subsequent activation of the PLCγ1, AKT and ERK1/2 pathways (**Fig. 2C**). We found that PLC γ1 had a higher level of activity in PKM2-depleted cells than in un-depleted cells after either a short or long (24 h) incubation with EGF. However, there was no marked difference in AKT activity between the PKM2-depleted cells and un-depleted cells. PLCγ is a key regulator of cell migration downstream of RTK signaling [11]. Phosphorylation on tyrosine residue 783 of PLCγ1 is critical to its activation [12]. PLCγ1 activation enhanced cell motility, and this effect was observed in the wound scratch and transwell assays, as observed in Fig. 1C.

We next investigated the effect of an EGFR ligand on the expression of MMPs using RT-PCR in BGC823-sipk and SGC7901-sipk cells compared with their respective control cells. Treatment with the EGFR ligand, EGF, enhanced the expression of MMPs at the level of transcription in BGC823 and SGC7901 cells. However, there were no obvious differences in the expression levels of MMP2 and MMP9 between PKM2-depleted cells and their control cells (data not shown). MMP7 expression was up-regulated in PKM2-depleted cells with EGF treatment (**Fig. 2D**). The ERK/MAPK pathways play critical roles in EGFR ligand-induced MMP7 expression. Furthermore, an obvious increase in ERK1/2 activity was observed after 0 h and 24 h of treatment with EGF in PKM2-depleted cells.

### Depletion of PKM2 Attenuated the Motility of AGS Cells and the Functional Changes after Rescuing PKM2 in Gastric Cancer Cell Lines

**Figure 3:**
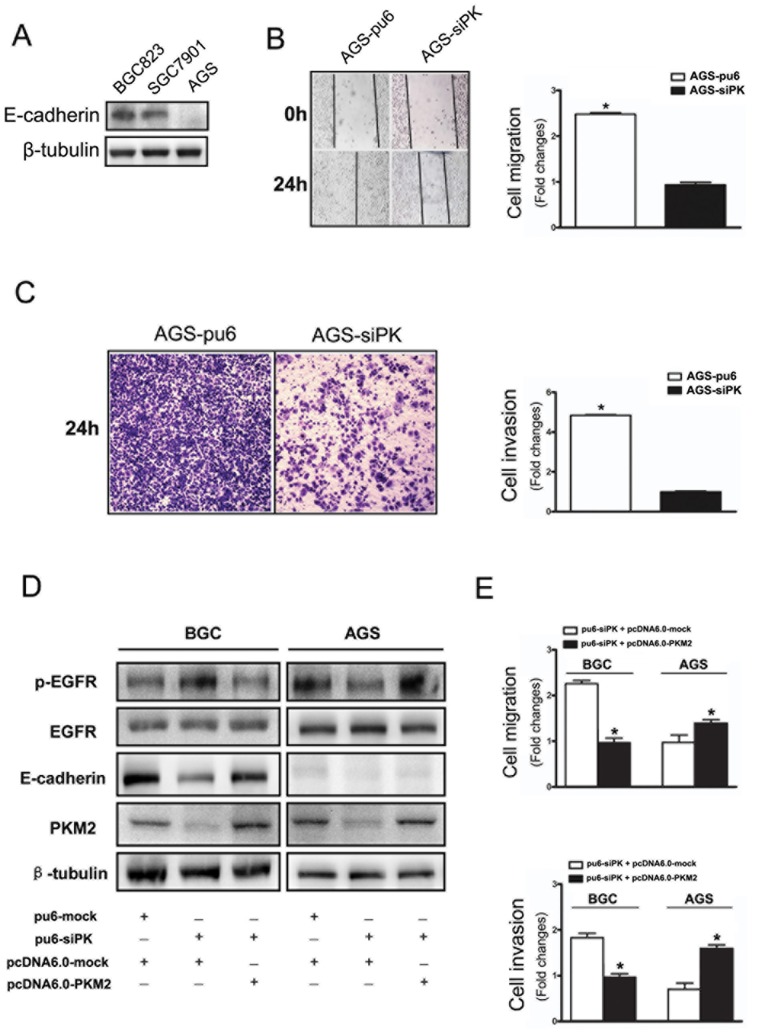
Depletion of PKM2 attenuated the motility of AGS cells and the functional changes after rescuing PKM2 in gastric cancer cell lines. (A) E-cadherin expression levels were detected by immunoblot analysis in BGC823, SGC7901 and AGS cells. (B) A cross-shaped wound was created in the monolayer, and the AGS stable cells were cultured for an additional 24 h with EGF (100 ng/ml). Representative images of the wounded region are shown. The results of the migration assay are also shown as graphs (*p<0.05). (C) The invasion potential of AGS stable cells was assessed using the BD transwell chamber assay with 100 ng/ml EGF in the lower chamber for 24 hours. The cells that migrated to the lower side of the filter were stained, photographed, and counted. (D) The expression of p-EGFR, E-cadherin in the PKM2 rescuing experiments with stably transfected method by using over-expression plasmid vector pcDNA6.0-mock and pcDNA6.0-PKM2 in BGC823 and AGS cells which stable knockdown PKM2. (E) The functional changes of cell migration and invasion after PKM2 rescuing. The data are expressed as the mean ± SD from three independent experiments (*p<0.05).

The expression of the E-cadherin protein in the gastric cancer cell lines BGC823, SGC7901 and AGS was evaluated with Western blot analysis. The BGC823 and SGC7901 cell lines express E-cadherin. In contrast, AGS cells lack E-cadherin expression (**Fig. 3A**).

To examine the effect of PKM2 knockdown on cell migration and invasion in AGS cells, we employed well-established wound-healing and transwell assays to characterize the cell motility. A confluent layer of cells was first incubated overnight in medium, a wound scratch was introduced, medium containing EGF (100 ng/ml) was added to stimulate migration, and the percentage of wound sealing was observed after 24 h. The invading cells in the transwell assay were quantified 24 h after EGF (100 ng/ml) was added to the lower chamber. To our surprise, we found that the treatment of AGS-sipk cells with EGF following the wound scratch and in the transwell significantly decreased the rate of wound sealing and invasion compared with that of the control cells (**Fig. 3B, C**). There were conspicuous differences between the BGC823/SGC7901 and AGS cells.

To further illustrate the role of PKM2 in cell motility, we did the PKM2 rescuing experiments. We taked stably transfected method by using over-expression plasmid vector pcDNA6.0-mock and pcDNA6.0-PKM2 to deal with BGC823 and AGS cells which stable knockdown PKM2. The expression of p-EGFR, E-cadherin were shown in the PKM2 rescuing experiments (Fig. 3D). We observed that when the PKM2 expression recovered, the phosphorylation of EGFR has significantly reduced in BGC823 cells and increased in AGS cells. Moreover, cell motility of BGC823 cells was decreased and AGS cells were declined after PKM2 rescuing (Fig. 3E). To clarify the mechanism of these differences, we then analyzed the activity of the EGF/EGFR signaling pathway.

### PKM2 Enhanced the Activities of the EGF/EGFR Downstream Signaling Pathways in AGS Cells and was Correlated with ERK Activity in Gastric Cancer Specimens

**Figure 4:**
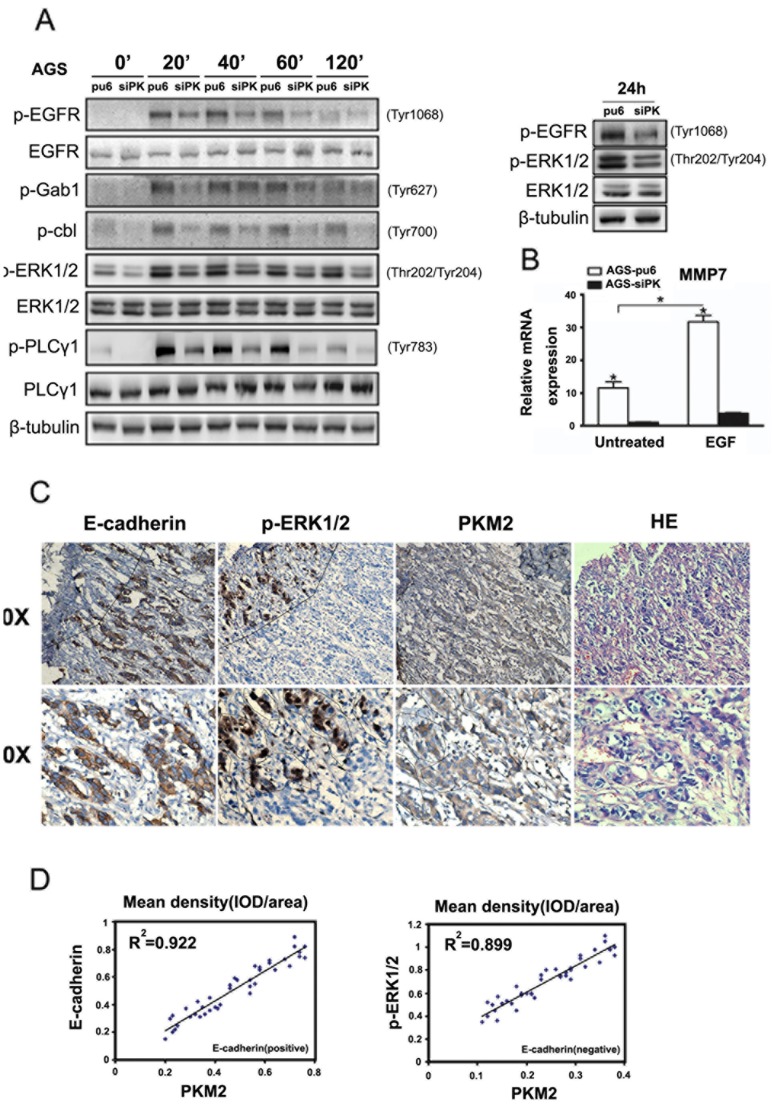
PKM2 enhanced the activities of EGF/EGFR downstream signaling pathways in AGS cells and was correlated with ERK activity in gastric cancer specimens. (A) AGS stable cells were exposed to EGF (100 ng/ml) for different times. Western blots of cell lysates were performed. The phospho-EGFR (Tyr1068), phospho-PLCγ1 (Tyr783), phospho-Gab1 (Tyr627), phosphor-c-cbl (Tyr700), and phospho-ERK1/2 (Thr202/Tyr204) protein levels are shown as indicated. (B) MMP7 expression levels were analyzed by quantitative real-time PCR in AGS stable cells. The data are expressed as the mean ± SD from three independent experiments (*p<0.05). (C) IHC staining with the indicated antibodies was performed on 15 human gastric cancer specimens. Representative photos of the tumor, which were taken in the same part of the same piece of tissue, are shown. (D) The correlation analysis among PKM2, E-cadherin and P-ERK1/2 was completed using the Image-pro Plus software. The mean density (IOD/area) was detected in different positive areas of 15 human gastric cancer specimens. The correlation analysis between PKM2 and E-cadherin was determined in an E-cadherin positive area. The correlation analysis between PKM2 and p-ERK1/2 was determined in an E-cadherin negative area.

To analyze whether the EGFR may be involved in the migration and invasion of AGS cells, these cells were treated with EGF, which binds to the EGFR and activates the downstream signaling pathways. EGF treatment resulted in the phosphorylation of the EGFR and the subsequent activation of the downstream EGFR pathways, including the PLCγ1 and ERK1/2 pathways (**Fig. 4A**). We found that the activities of PLCγ1 and ERK1/2 were greater in cells where PKM2 was not depleted than in the PKM2-depleted cells after either a short or long (24 h) incubation with EGF. This result is the opposite of what was observed with the BGC823 and SGC7901 cells; in AGS cells, PKM2 came into play as a stimulus and promoted cell migration and invasion. We next investigated MMP7 expression using RT-PCR in AGS-sipk cells and the control cells. Treatment with EGF enhanced MMP7 expression at the level of transcription in AGS-pu6 cells but not in AGS-sipk cells (**Fig. 4B**). The activity of ERK1/2 was obviously higher in AGS-pu6 cells compared with AGS-sipk cells after 0 h and 24 h treatment with EGF (**Fig. 4A**).

We next performed immunohistochemical (IHC) analyses to examine E-cadherin expression, PKM2 localization and ERK1/2 phosphorylation in serial sections of 15 human gastric cancer specimens using antibodies with validated specificities. Figure 4C shows that the levels of E-cadherin expression, ERK1/2 phosphorylation, and cytoplasmic PKM2 expression were correlated with each other. In addition, we observed a high level of ERK1/2 phosphorylation in the nucleus of cancer cells without E-cadherin expression. In areas of ERK1/2 phosphorylation, we also found higher levels of PKM2 expression. However, we did not find the phosphorylation of ERK1/2 in areas positive for E-cadherin expression (**Fig. 4C**). A correlation analysis among PKM2, E-cadherin and P-ERK1/2 was performed using Image-pro Plus software (**Fig. 4D**). The mean density (IOD/area) was recorded in different positive areas of 15 human gastric cancer specimens. We found a significant correlation between PKM2 and E-cadherin in E-cadherin-positive areas. Moreover, there was a significant correlation between PKM2 and p-ERK1/2 in E-cadherin-negative areas.

## Discussion

The invasive and metastatic stage of cancer progression correlates with poor clinical prognosis and represents the most formidable barrier to successful treatment. Cell motility and invasiveness are the defining characteristics of malignant tumors, which enable tumor cells to migrate into adjacent tissues or through limiting basement membranes and extracellular matrices. Cell motility is required for the physiological processes of wound repair and organogenesis and for the pathological process of tumor invasion [13]. Invasive tumor cells are characterized by dysregulated cell motility in response to extracellular signals from growth factors and cytokines. Human tumors express high levels of growth factors and their receptors, and many types of malignant cells appear to exhibit autocrine- or paracrine-stimulated growth. Among the most well-studied growth factor receptor systems is the EGF receptor family [14]. Signals from the extracellular milieu dictate cell motility. Many growth factors, including the ligands that act through the epidermal growth factor receptor (EGFR), enhance cell motility [15]. At least two distinct intracellular signaling pathways are required for EGFR-mediated cell motility: the pathways utilizing PLC γ and the MAP kinase pathway. PLC γ activity has been proposed to enhance cell motility through the mobilization of actin-modifying proteins from an inactive membrane-associated localization to an active sub-membrane cytoskeletal locale [16]. The Erk MAP kinases transmit signals to the nucleus as well as signals that regulate cell-matrix connections.

Cadherins comprise a large family of cell–cell adhesion molecules that include the classical, desmosomal, and atypical cadherins. E-cadherin, which is expressed primarily in epithelial cells, is an adhesion protein that is encoded by the CDH1 gene and functions in multiple processes, including development, tissue integrity, cell migration, morphology, and polarity [17], [18], [19]. E-cadherin is also a tumor suppressor whose expression is frequently reduced or silenced, and its re-expression can induce morphologic reversion [20], [21]. The EGF-dependent activation of the EGFR has been reported to be inhibited in an E-cadherin adhesion-dependent manner, which inhibits the ligand-dependent activation of diverse receptor tyrosine kinases. N-cadherin, as an invasion promoter, is frequently upregulated. The expression of N-cadherin in epithelial cells induces changes in morphology to a fibroblastic phenotype, rendering the cells more motile and invasive. Recent studies indicate that cancer cells have up-regulated N-cadherin in addition to the loss of E-cadherin. This change in cadherin expression is called the “cadherin switch”.

We observed a down-regulation of E-cadherin mRNA and increased phosphorylation, which induces the endocytosis of E-cadherin, in PKM2-depleted cells. We also found that the N-cadherin protein expression level was increased in the BGC823 cell line when PKM2 was depleted. The knockdown of PKM2 promoted cell migration and invasion in BGC823 and SGC7901 cells with EGF stimulation. An increased activity of the EGFR is the critical factor in determining the cell motility and invasiveness of BGC823 and SGC7901 cells. We hypothesized that the down-regulation of E-cadherin expression enhanced the phosphorylation of the EGFR and activated the downstream signaling pathways, which include PLCγ1 and ERK1/2. PLC γ activation enhances cell motility, and ERK1/2 activity plays a critical role in MMP7 expression.

Matrix metalloproteinases (MMPs) have been implicated in cancer invasion and metastasis because of their extracellular matrix (ECM)-proteolytic activity [22]. MMP-7 has been reported to be produced by gastric carcinoma cells and is significantly associated with the aggressive pathological phenotypes of gastric cancer [23]. MMP-7 can activate pro-MMP-1, has strong stromelysin-like activity and degrades insoluble elastin, type IV collagen, laminin-1, fibronectin, proteoglycan and gelatins [24]. The ERK/MAPK pathways play critical roles in EGF–induced MMP7 expression [25].

E-cadherin is a cell-adhesion glycoprotein characterized for the first time in human cell lines by Shimoyama et al [26]. Its role in gastric cancer development was first defined by Guilford et al [27]. There is a significant correlation between the degree of E-cadherin expression and the grade of tumor differentiation, as well as the histological type according to the Laurén and the WHO classifications. Patients with E-cadherin-positive tumors have significantly better 3- and 5-year survival rates than patients with E-cadherin-negative tumors [28]. Hereditary diffuse gastric cancer (HDGC) is a rare autosomal dominant syndrome that is largely attributable to germline mutations and deletions in the CDH1 gene associated with an early onset, histologically diffuse, signet-ring cell type gastric cancer [29], [30].

Lim JY et al reported that PKM2 expression was strongly correlated with gastric cancer differentiation. Differentiated types of cancers express more PKM2 protein than the undifferentiated types; in contrast, higher PKM2 expression is correlated with shorter overall survival independent of stage in signet-ring cell cancers. PKM2 expression might be an adverse prognostic factor for signet-ring cell carcinomas, which lack E-cadherin [7]. These results are in accordance with our research in gastric cancer cells. The BGC-823, SGC-7901 and AGS cell lines are differently differentiated types. E-cadherin expression exists in the SGC-7901 and BGC-823 cell lines; in contrast, the AGS cells were derived from malignant gastric adenocarcinoma tissue and lack E-cadherin-mediated cell adhesion [31]. We observed that the knockdown of PKM2 promoted the migration and invasion of the SGC-7901 and BGC-823 cell lines but suppressed these properties in the AGS cell line. Another group has reported that pyruvate kinase type M2 is upregulated in colorectal cancer, and the knockdown of PKM2 suppressed the proliferation and migration of colon cancer RKO cells [32]. We know that RKO cells lack the expression of E-cadherin [33]. Immunohistochemical (IHC) analysis demonstrates that the levels of E-cadherin expression, ERK1/2 phosphorylation, and cytoplasmic PKM2 expression were correlated with each other. We found a high level of ERK1/2 phosphorylation in the nucleus of cancer cells without E-cadherin expression but with a high level of PKM2 expression.

We hypothesize that PKM2 attenuates cell motility and invasion when E-cadherin is present. This novel function of PKM2 may play a role in the reversible inhibition of cell motility and invasion in the early stages of gastric cancer when cells are positive for E-cadherin expression. During the progression of the tumor, a lack of or very low expression of E-cadherin induces an aggressive function of PKM2 in the tumor. The biological role of PKM2 in the development of these tumors must be further elucidated.

## Supporting Information

Figure S1 **The expression of the EGFR protein in the gastric cancer cell lines BGC823, SGC7901 and AGS was evaluated using Western blot analysis.** AGS cells showed a higher level of EGFR expression than the other two cell lines. There is no significant difference between BGC823 and SGC7901 cells (Figure S1A). BGC-pu6 cells and BGC-sipk cells were treated with different doses of EGF. After 40 minutes we detected the level of phosphorylation for EGFR. We found the highest level of phosphorylation in the dose of 100ng/ml (Figure S1B). Therefore we chose the dose of 100ng/ml as the most suitable candidate. The transwell experiment also showed the stronger ability to penetrate the martrigel in BGC823 cells (Figure S1C).
(TIF)
