# Supplementary figures and images for: Pyruvate Kinase M2 Plays a Dual Role on Regulation of the EGF/EGFR Signaling via E-Cadherin-Dependent Manner in Gastric Cancer Cells
Source: PLoS One. 2013 Jun 28;8(6):e67542. doi: 10.1371/journal.pone.0067542 (PMC3695906; doi:10.1371/journal.pone.0067542)

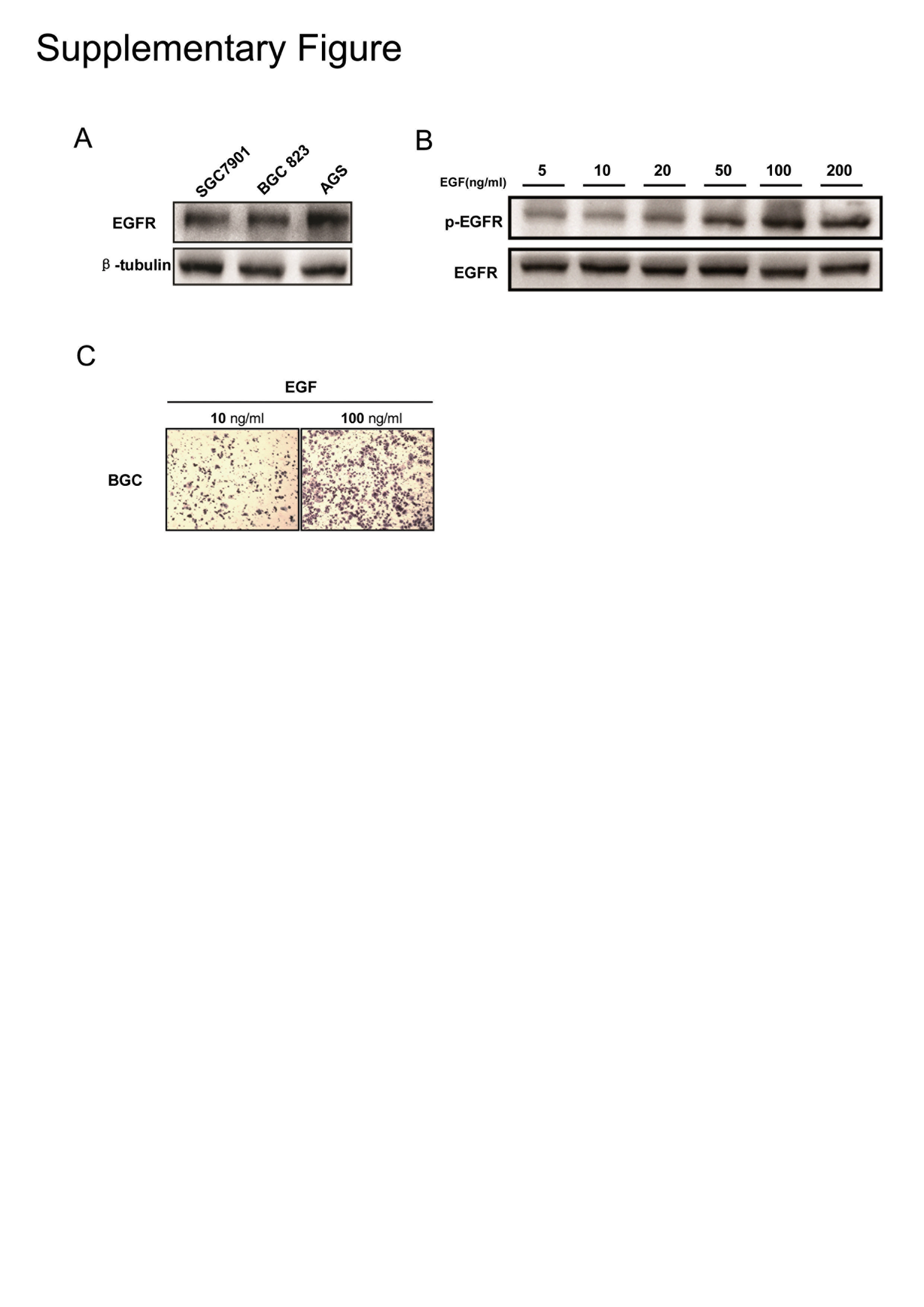

Supplement: Figure S1 — The expression of the EGFR protein in the gastric cancer cell lines BGC823, SGC7901 and AGS was evaluated using Western blot analysis. AGS cells showed a higher level of EGFR expression than the other two cell lines. There is no significant difference between BGC823 and SGC7901 cells (Figure S1A). BGC-pu6 cells and BGC-sipk cells were treated with different doses of EGF. After 40 minutes we detected the level of phosphorylation for EGFR. We found the highest level of phosphorylation in the dose of 100ng/ml (Figure S1B). Therefore we chose the dose of 100ng/ml as the most suitable candidate. The transwell experiment also showed the stronger ability to penetrate the martrigel in BGC823 cells (Figure S1C). (TIF) [file pone.0067542.s001.tif]
